# Evaluating the Impact of Occlusal Adjustment on Post-Orthodontic Outcomes: Insights from T-Scan III Analysis 

**DOI:** 10.30476/dentjods.2025.105593.2604

**Published:** 2026-06-01

**Authors:** Arezoo Jahanbin, Maryam Omidkhoda, Farnaz Zia, Azam Sadat Madani, Ali Koohrokhi, Zahra Hosseini

**Affiliations:** 1 Dept. of Orthodontics, School of Dentistry, Mashhad University of Medical Sciences, Mashhad, Iran.; 2 Dept. of Orthodontics, School of Dentistry, Guilan University of Medical Sciences, Rasht, Iran.; 3 Dept. of Prosthodontics, School of Dentistry, Mashhad University of Medical Sciences, Mashhad, Iran.; 4 Postgraduate Student, Dept. of Prosthodontics, School of Dentistry, Mashhad University of Medical Sciences, Mashhad, Iran.; 5 Postgraduate Student, Dept. of Orthodontics, School of Dentistry, Mashhad University of Medical Sciences, Mashhad, Iran.

**Keywords:** Orthodontics, Occlusal Adjustment, Dental occlusion, T-scan

## Abstract

**Background:**

Occlusal changing, spacing, and tooth movement are common problems caused by premature contact after orthodontic treatment. Occlusal adjustment has been suggested to help stabilize orthodontic treatment. T-Scan III system is a rapid and accurate diagnostic tool for digital occlusal analysis in different fields of dentistry, particularly in orthodontics.

**Purpose:**

This study aimed to evaluate the impact of occlusal adjustment on post-orthodontic occlusal parameters using the T-Scan III system, focusing on force distribution and occlusal contact dynamics.

**Materials and Method:**

This interventional randomized controlled trial included 30 patients (17 females and 13 males) aged 17-28 years with Class I malocclusion and severe crowding. The patients were divided into intervention and control groups. All patients under-went fixed orthodontic treatment with the extraction of the first four premolars. The intervention group received occlusal adjustments one month after debonding, while the control group received no intervention. Occlusal relationships were recorded at debonding (T0), one month (T1), and six months (T2) post-debonding using the T-Scan III system. The analyzed key parameters included the occlusal contact intensity, number, and distribution. Independent and paired t-tests, the Mann-Whitney test, the Chi-square test, and the Wilcoxon statistic were used. The significance level for statistical tests was set at *p* Value< 0.05.

**Results:**

No statistically significant differences were observed between the groups in occlusal force intensity or number of contacts at T1 and T2. However, these trends suggest an improved posterior force distribution in the intervention group, highlighting the clinical benefits of the occlusal adjustment.

**Conclusion:**

Although not statistically significant, the results suggest that occlusal adjustments supported by T-Scan III analysis may enhance occlusal stability and force distribution after orthodontic treatment. Future studies with larger cohorts and longer follow-up periods are recommended to validate these findings.

## Introduction

Occlusion refers to static contact between the upper and lower teeth and is influenced by a variety of dynamic factors [ 1 ]. The temporomandibular joint (TMJ) and muscles control mandibular motion and interact with opposing occlusal surfaces to determine the occlusion [ 2 ]. Understanding the various aspects of occlusion, both before and after treatment, is critical for making informed choices and achieving optimal orthodontic treatment [ 3 ].

Occlusal adjustment, which is frequently performed following restorative procedures, is usually a trial-and-error approach that focuses on patient comfort over masticatory function. The interaction between the teeth and the entire masticatory system, which begins at the TMJ, is the only way to understand effective occlusal adjustments [ 4 ]. Accurate correction is impossible without acknowledging that the main objective of occlusal adjustment is to remove occlusal interference, which impairs the physiological function of the joint. Occlusal adjustment techniques include the removal of premature contacts in centric relation (CR), elimination of interferences to align maximum intercuspation position (MIP) with centric relation, and removal of premature contacts on the non-working side [ 4 - 5 ].

Orthodontic treatment aims to restore normal occlusion by altering occlusal relationships. Numerous quantitative occlusal indicators have been used, including alginate impression materials, silicone putty, polyether, articulating paper, silk strips, Shimstock films, occlusal sprays, and occlusal ultrasonography, each with its own advantages and disadvantages [ 6 ]. The use of digital technologies, such as the T-Scan III system, is increasingly favored because of evidence that traditional methods often fail to capture peak bite force with precision [ 7 ].

T-Scan III technology includes a sensor that detects the relative intensity and timing of occlusal contacts, providing detailed information about pressure distribution across the dental arches [ 8 ]. Lin's concordance correlation coefficient (CCC) and intra-class correlation coefficient (ICC) have been used to demonstrate the T-Scan III system's great reliability for successive readings [ 9 ]. This method evaluates the entire dental system by analyzing the force distribution in the anterior, premolar, and molar areas, and comparing the left and right sides of the jaw. It detects peak contacts, tooth-type contacts, mandibular deviations, occlusion timing, and contact discrepancies between the working and nonworking sides [ 7 ].

Given that digital technology has entered many scientific fields, improving precision and repeatability has greatly decreased clinician time requirements. Consequently, digital approaches have recently received considerable attention. Digital technology has advanced both qualitative and quantitative assessments of occlusal contacts in dental clinics, particularly in prosthodontics, restorative, and orthodontic care [ 10 ]. 

Although minor post-treatment occlusal changes are expected, they can cause significant functional problems if they remain unaddressed. Conventional orthodontic indices mainly assess tooth alignment and overlook the timing and force of the occlusal contact. However, even slight premature contact or uneven force can lead to functional imbalance, TMJ strain, or chewing issues [ 11 ]. Studies using T-Scan III show that clinically acceptable occlusions can still be functionally unstable; digitally guided finishing improves long-term balance [ 12 - 14 ]. Furthermore, even minor occlusal imbalance is linked to TMJ issues during retention and should be digitally monitored [ 11 ]. 

Given the scarcity of research on occlusal adjustment following orthodontic treatment, this study was conducted to determine its impact on occlusal status, as assessed by the T-Scan III system. Our findings underscore the clinical value of digital occlusal analysis in detecting subtle functional imbalances, with the potential to enhance treatment outcomes and long-term stability.

## Materials and Method

This interventional clinical trial was approved by the Ethics Committee of the Mashhad University of Medical Sciences (IR.MUMS.DENTISTRY.REC.1401.143) and registered in the Iranian Registry of Clinical Trials ( IRCT20230310057665N1). The Consolidated Standards of Reporting Trials (CONSORT) guidelines and *Helsinki Declaration* principles were followed in this study. All participants provided written informed consent before enrollment.

### Sample size

In a pilot study with five patients in each group, the greatest pre- and post-treatment score difference was observed in the variable intermolar distance (IMD), with values of 1.95±1.80 in the intervention group and -1±2.5 in the control group. These results were used to calculate the sample size, which was calculated with a 95% confidence level, 80% power, and 10 patients per group, based on the pilot study findings. To ensure reliability, the sample size was increased to 15 patients per group, resulting in 30 patients in both the study groups. Although statistically justified, the small pilot sample may have limited the accuracy of variance estimation, potentially affecting the study's power to detect subtle differences. 

### Study design

All patients of both sexes aged between 17 and 28 years, with Class I dental malocclusion and crowding exceeding 8mm, who needed fixed orthodontic treatment involving the extraction of the four first premolars in both arches and were treated using the MBT 0.022 prescription system. All patients were referred to the Orthodontics Department of Mashhad Dental School. Exclusion criteria were systemic diseases, poor oral hygiene, gingivitis, periodontitis, deep caries extending to the cementoenamel junction, use of medications affecting bone metabolism, such as antiresorptive drugs, presence of parafunctional habits, and a history of TMJ disorders.

The patients were divided into two groups: control and intervention (n=15). At the end of orthodontic treatment, the bands and brackets were removed, the composite and cement were removed, and scaling was performed. Occlusal relationships were recorded with T-scan III (Tekscan; Inc., South Boston, MA, USA) at three intervals: immediately after debonding (T0), one month (T1), and six months (T2) post-debonding in centric relation.

In both groups, the patients received an upper and lower Essix retainer within a maximum of one week. The retainers were made of 1.5mm thick transparent thermoplastic sheets (Crystal® Plate, Bio Art Dental Equipment Ltda., São Carlos/SP, Brazil), composed of PETG (polyethylene terephthalate glycol), a material commonly used for fabricating removable retainers. They covered 1-2 mm of the buccal and 3-4 mm of the palatal surface, respectively. In the first four months, the retainer was placed throughout the day, except for eating and brushing teeth, and then only at night. Fixed retainers were not used in either group to avoid interference in the analysis of occlusal contact changes. During the one-month follow-up, occlusal adjustment was performed in the intervention group but not in the control group. Participants in both groups were evaluated one- and six-month post-treatment. Occlusal relationships were recorded using the T-Scan system in centric relation. At debonding (T0), all the patients exhibited acceptable static intercuspation. While specific occlusal patterns, such as canine guidance and group function, were not clinically classified, the T-Scan III data confirmed comparable anterior and posterior contact distributions between groups, indicating similar baseline occlusal status. This six-month period was chosen to capture the early functional changes after debonding and occlusal adjustment. We acknowledge that a 12-month period or longer could provide additional insights into long-term occlusal stability and recommend this for future studies.

### Occlusal adjustment 

Occlusal adjustment was performed by an orthodontics post-graduate student under the supervision of a specialist. The muscles were deprogrammed using cotton rolls, and centric relation was achieved using the Dawson approach, a method used to establish a stable and healthy relationship between the upper and lower jaws by focusing on centric relation. The centric relation is the position where the jaw joints (temporomandibular joints, TMJ) are in their most relaxed and stable state, with the condyles of the lower jaw resting in their optimal position within the joint sockets.

In this approach, the muscles are first deprogrammed to ensure relaxation, typically by having the patient bite on soft materials, such as cotton rolls. This eliminates muscle tension that could interfere with finding the correct jaw position. The operator placed four fingers along the inferior border of the mandible, with the pinky supporting the mandibular angle, and both thumbs resting lightly over the symphysis in a “C” shape. The mandible was gently manipulated through hinge-like opening and closing movements until it was seated in CR without resistance. Once CR is achieved, occlusal contacts are identified using tools like 8-micron thickness articulation paper (Arti-Fol, Germany) to mark are-as where teeth meet prematurely. These interferences were subsequently adjusted to create even and balanced contact across the dental arches. The objective is to align the jaw and teeth to minimize strain on the TMJ, prevent tooth damage, and ensure efficient and comfortable chewing. Heavy contacts were defined as those producing thick, continuous markings on the articulating film and confirmed by T-Scan III recordings showing a contact force above 20% of the total occlusal load. The average enamel reduction per adjustment ranged from 50 to 100 microns. Heavy contacts were removed using egg-shaped 833F (Jota, Switzerland) and high-speed handpieces. The occlusion correction continued until all tips of the centric cusps made contact with the opposite fossa and the mandible remained unchanged. In the second stage, eccentric interference was examined and corrected in lateral and forward movements. All interference on the working and balancing sides was removed to achieve canine guidance. Fluoride therapy was administered to prevent tooth sensitivity in all patients.

### Occlusal scanning

The sensor was positioned identically across all sessions to ensure consistent and reliable T-Scan recordings. Patients were seated with the Frankfort plane parallel to the ground, and the mandible was guided to centric relation using a physiological swallowing technique, in which the patient was told to bring his tongue to the back of his palate and swallow his saliva. Each sensor was made of 85-micron-thick polyester and included 2,500 pressure-sensitive cells, which recorded occlusal contact when the patient bit down. The anterior part of the sensor was placed on the midsagittal line of the sheet between the two centers. The patient closed her/his mouth in the same manner, three times in a row. To prevent muscle fatigue, the patient was given a 3-minute break between bites. The mean value was used for analysis. All recordings were performed by a single trained operator to eliminate inter-examiner variability. 

**Figure 1:**
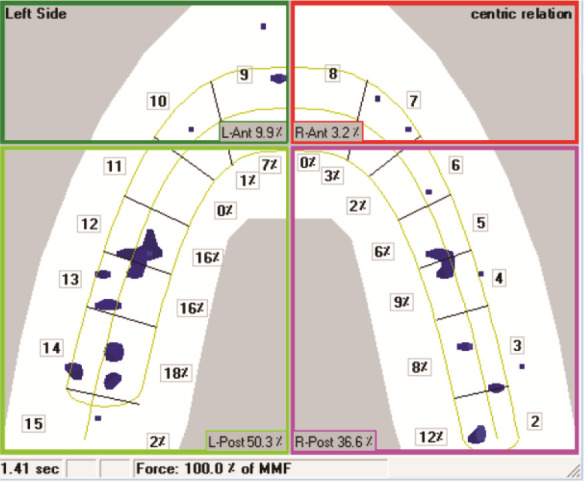
Occlusal diagram of the contact points’ distribution, recorded from the T-Scan device

This software captured the location, number, and strength of occlusal contacts in both 2D and 3D images. The software properly distributed the occlusal force across the entire jaw arch; the time elapsed between the first contact and maximum intercuspation, and the jaw pathway during mouth closure. Figure 1 shows a graph recorded by the system that displays the time elapsed between the first contact and maximum intercuspation. The green color represents the biting force on the left side of the jaws, and the red color on the right. 

### Data Analyze

The data were analyzed using SPSS version 25 (SPSS Inc., Chicago, IL, USA). Independent and paired t-tests, the Mann-Whitney test, the Chi-square test, and the Wilcoxon statistic were used. The significance level for statistical tests was set at *p*< 0.05. 

## Results

This study included 30 patients, comprising 17 women (56.7%) and 13 men (43.3%), all of whom underwent orthodontic treatment. The average age was 20.20±3.20 years. The age range of each group was 17-28 years old. The intervention group had an average age of 20.57± 3.45 years, while the control group had an average of 19.83±3.00 years. This difference was not statistically significant (*p*= 0.624). The intervention and control groups had 8 (53.3 percent) and 9 (60 percent) women, respectively, and 7 (46.7 percent) and 6 (40 percent) men, respectively. In general, there were no significant differences between the groups (*p*= 0.713).

To examine occlusal contacts, the data distribution was verified for normality using the Shapiro-Wilk test, which revealed that all data were normally distributed. 

Table 1 shows the number; meanSD, minimum and maximum values ​​of occlusal contact intensity by group, and the results of statistical tests. Occlusal contact intensity and the number of occlusal contacts were each measured three times in the anterior and posterior regions on both sides, and the results were categorized by tooth type (molar, premolar, central, lateral, and canine) within the intervention and control groups. For this reason, the distribution of the quantitative variables was tested for normality. The independent t-test was used for normal data, whereas the Mann-Whitney test was used for non-normal data. Table 2 shows the number, mean SD, minimum and maximum values ​​of occlusal contact number, and also the results of statistical tests. 

**Table 1:** Comparison of changes in the intensity of occlusal contact between the two groups

Data	Group	Number	MeanSD	Max	Min	T-test
T1-T0 difference in the anterior region	2	15	-1.43±5.43	9.50	-10.50	T= 0.99
	1	15	0.60±5.78	12.50	-10.50	*p*= 0.329
T1-T0 difference in the posterior region	2	15	1.10±6.10	10.50	-12.00	T= 0.78
	1	15	-0.60±5.78	10.50	-12.50	*p*= 0.440
T2-T0 difference in the anterior region	2	15	-2.17±6.34	8.00	-13.00	T= 1.23
	1	15	0.23±4.15	7.50	-6.50	*p*= 0.230
T2-T0 difference in the posterior region	2	15	2.17±6.34	13.00	-8.00	T= 1.23
	1	15	-0.23±4.15	6.50	-7.50	*p*= 0.230
T2-T1 difference in anterior region	2	15	-0.73±4.42	7.00	-7.50	T= 0.24
	1	15	-0.37±4.08	6.50	-6.00	*p*= 0.815
T2-T1 difference in the posterior region	2	15	1.07±4.51	7.50	-7.00	T= 0.45
	1	15	0.37±4.08	6.00	-6.50	*p*= 0.659

T0: Immediately after treatment, T1: one-month follow-up, T2: six-month follow-up; Group 1: Control, Group 2: Intervention

**Table 2:** Comparison of changes in the number of occlusal contacts between the two groups

Data	Group	Number	MeanSD	Max	Min	Test
T1-T0 difference in the anterior region	2	15	-0.600±1.638	2.00	-3.00	T=- 0.429
	1	15	0.133±1.125	2.00	-2.00	*p*= 0.164
T1-T0 difference in posterior region	2	15	1.33±1.345	4.00	-1.00	T= -1.192
	1	15	0.466±2.474	6.00	-4.00	*p* =0.243
T2-T0 difference in the anterior region	2	15	-0.266±1.667	2.00	-2.00	T= -0.248
	1	15	-0.133±1.245	2.00	-2.00	*p* =0.806
T2-T0 difference in posterior region	2	15	0.733±1.980	5.00	-1.00	Z= -0.127
	1	15	0.466±1.959	4.00	-3.00	*p*= 0.902
T2-T1 difference in anterior region	2	15	0.333±1.632	5.00	-2.00	Z=-1.226
	1	15	-0.266±0.883	2.00	-1.00	*p*= 0.250
T2-T1 difference in posterior region	2	15	0.483±1.956	4.00	-5.00	Z=-1.097
	1	15	0.000±1.690	3.00	-3.00	*p*= 0.285

T0: Immediately after treatment, T1: one-month follow-up, T2: six-month follow-up; Group 1: Control, Group 2: Intervention; Z: Mann-Whitney test result

## Discussion

This study evaluated the role of digital occlusal analysis using the T-Scan III system in assessing occlusal adjustment after orthodontic treatment. Occlusal harmony is critical for ensuring functionality, stability, and long- term success of orthodontic outcomes. The recurrence of primary malocclusion and disruption of jaw relations have been considered among the most challenging challenges in orthodontic treatment since its inception [ 15 ]. This issue often causes concern among patients, even if not all of them explicitly express it. Several prior studies have highlighted the importance of using dynamic digital occlusal indicators such as the T-Scan system, which provides precise quantitative data on occlusal forces and their distribution [ 10 , 16 ]. 

Occlusal adjustments aim to optimize force distribution and eliminate interferences that could compromise the TMJ or overall functionality [ 17 ]. Research has shown that aligning CRs with MIP enhances both patient comfort and the overall success of treatment outcomes. Furthermore, ensuring balanced occlusal contact reduces the risk of periodontal strain and long-term relapse [ 18 ].

Compared to traditional techniques such as articulating paper, the T-Scan III system offers substantial bene fits, including the ability to assess occlusal force distribution, timing of contacts, and symmetry in real time [ 10 , 16 ]. Unlike static tools, T-Scan provides dynamic recordings, offering insights into the influence of occlusal adjustments on mandibular movement and joint health. Furthermore, digital occlusal analysis using T-Scan III has proven to be invaluable in identifying and addressing subtle interferences. The ability of the system to measure and visualize occlusal forces, time to maximum intercuspation, and contact dynamics supports its superiority over conventional methods, such as articulating paper [ 2 ].

Although T-Scan III sensors primarily measure relative occlusal forces, they demonstrate high intra-session reliability when used under standardized conditions [ 9 ].

For this study’s purpose which is evaluating temporal changes in occlusal force distribution rather than absolute force, the system’s built-in calibration and relative force output were sufficient and consistent [ 9 ]. The results of this study revealed no statistically significant differences in the force distribution or contact number changes between the intervention and control groups.

Similar non-significant outcomes have been reported in the literature. Bonadio *et al*. [ 17 ] found no difference in occlusal stability or relapse between patients who received occlusal adjustment and those who did not, despite long-term follow-up. Likewise, Fathalla *et al*. [ 3 ] observed no statistically significant changes in bite force distribution before and after orthodontic treatment with premolar extractions, despite improvements in posterior force balance. However, the trend of improved posterior balance in the intervention group underscores the clinical benefits of occlusal adjustment. This finding aligns with prior research emphasizing the need for balanced posterior forces to minimize masticatory strain and prevent TMJ-related issues [ 5 , 13 ]. 

The study by Bonadio *et al*. [ 17 ] included 77 orthodontic patients with Class I malocclusion that did not require tooth extraction. The retention procedure included a Hawley retainer in the maxilla and a canine-to-canine bonded retainer in the mandible. In the intervention group, Little's irregularity index and peer assessment rating (PAR) index were assessed before treatment, immediately following debonding, and 5 years later using casts obtained from patients in both groups. Little's irregularity index and PAR Index did not differ between the intervention and control groups in any phase.

In a study by Koval *et al*. [ 19 ], a 29-year-old woman with anterior tooth crowding and Class I skeletal, molar, and canine relationships underwent orthodontic treatment for 23 months. A canine-to-canine bonded retainer was placed in the patient's upper and lower jaws, and a T-scan was performed. During the first contact cycle, the right molar teeth had maximum pressure, accounting for 30% of the total pressure. Significant occlusal contacts in the area of the right first and left second molars were identified and treated using occlusal foils, resulting in a shorter primary closing cycle and resolution of the overload. In addition, the contacts were not linear during lateral movements but became linear when the occlusal position was found. Finally, occlusal contacts at the end of orthodontic therapy require occlusal adjustments to minimize occlusal overload and achieve bilateral balance [ 19 ]. In the present study, occlusal adjustment was used to manage severe occlusal contacts, and the results were examined using a T-scan immediately after adjustment. The results were satisfactory, indicating that the posterior occlusal contacts had become more severe; nevertheless, after six months, due to continuing tooth settling, we found different situations.

Mahmoud Helal *et al*. [ 20 ] used T-scan III to analyze the distribution of contacts in different movements between patients who received orthodontic therapy without tooth extraction and those who did not get orthodontic treatment. The orthodontic group had heavier dental contacts in the central occlusion, and there was no difference in the lateral movement on the working side between the two groups. Premature contacts were on the premolars on the balancing side in the intervention group and on the molars in the control group. This study concluded that tooth contact following orthodontic treatment differed from that of individuals who had never received such treatment, with the orthodontic group exhibiting more premature contacts on both the centric occlusion and balancing sides. The adoption of digital tools such as T-Scan III has revolutionized occlusal analysis by offering precise, repeatable data on contact intensity and distribution. Studies have highlighted the ability of this system to dynamically track force imbalances and provide real-time feedback, facilitating more accurate adjustments and improving patient outcomes [ 11 - 12 ]. Additionally, this technology reduces the treatment time, enhances precision, and minimizes subjective variability in occlusal assessments [ 21 ].

While the sample size was determined based on pilot data and statistical power calculations, we acknowledge this as a limitation and suggest that future studies should include larger cohorts to enhance external validity. 

Although this study provides valuable insights, its relatively short follow-up period may have limited the statistical significance of its findings. The focus on a specific orthodontic subgroup also limits broader generalization. Future research with larger, more diverse populations and extended follow-up alongside patient-reported outcomes can help validate and expand upon these findings. 

## Conclusion

While occlusal adjustment did not demonstrate a statistically significant improvement in occlusal stability within the six-month follow-up, the trends suggest that occlusal adjustments supported by digital technologies, such as T-Scan III, help refine occlusal contact relationships, enhance post-orthodontic stability and highlight the potential benefits of integrating digital occlusal analysis into routine orthodontic practice. Larger studies with extended follow-ups are recommended to clarify the long-term benefits of occlusal adjustment in post-orthodontic care.
